# In Silico Prediction of Drug-Induced Liver Injury Based on Ensemble Classifier Method

**DOI:** 10.3390/ijms20174106

**Published:** 2019-08-22

**Authors:** Yangyang Wang, Qingxin Xiao, Peng Chen, Bing Wang

**Affiliations:** 1 Institutes of Physical Science and Information Technology, Anhui University, Hefei 230601, China; 2 School of Computer Science and Technology, Anhui University, Hefei 230601, China; 3 School of Electrical and Information Engineering, Anhui University of Technology, Ma’anshan 243032, China

**Keywords:** drug-induced liver injury, quantitative structure–activity relationship (QSAR), molecular fingerprints, ensemble classifier

## Abstract

Drug-induced liver injury (DILI) is a major factor in the development of drugs and the safety of drugs. If the DILI cannot be effectively predicted during the development of the drug, it will cause the drug to be withdrawn from markets. Therefore, DILI is crucial at the early stages of drug research. This work presents a 2-class ensemble classifier model for predicting DILI, with 2D molecular descriptors and fingerprints on a dataset of 450 compounds. The purpose of our study is to investigate which are the key molecular fingerprints that may cause DILI risk, and then to obtain a reliable ensemble model to predict DILI risk with these key factors. Experimental results suggested that 8 molecular fingerprints are very critical for predicting DILI, and also obtained the best ratio of molecular fingerprints to molecular descriptors. The result of the 5-fold cross-validation of the ensemble vote classifier method obtain an accuracy of 77.25%, and the accuracy of the test set was 81.67%. This model could be used for drug-induced liver injury prediction.

## 1. Introduction

**Figure 1:**
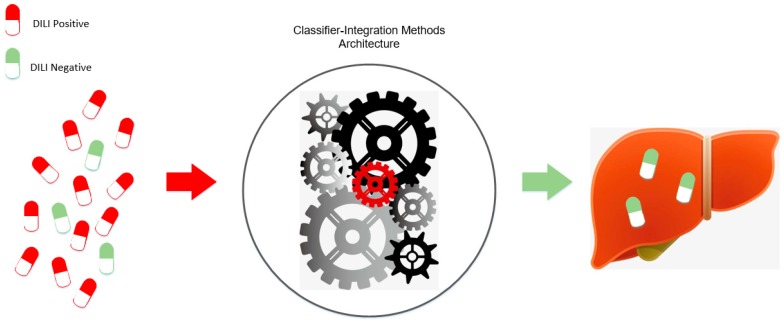
Concept map of drug-induced liver injury (DILI) modeling process.

New drug development was affected by many factors [1], which made 90% potential drugs failing in the clinical trial phase [2]. Previous studies showed that drug efficacy and toxicity are the two main causes of drug development failure [3], in which liver damage is the most important cause [4]. Therefore, in the practice of clinical medication, prevention of drug-induced liver injury is one of the most important issues [5]. In the development of drugs, it is important to screen for effective compounds in the early stages of drug development, and to exclude compounds with drug-induced liver damage characteristics. In the past decades, many works have presented a number of methods to assess the risk of drug-induced liver injury, however they are time-consuming and labor-intensive, and always yielded unsatisfactory results [6]. Figure 1 shows the concept map of DILI modeling process.

In recent years, many machine learning methods have made great contributions to the prediction of hepatotoxicity [7], especially the QSAR model, which has been widely used in liver toxicity research [8]. Machine learning modeling for the prediction of DILI [9] was based on the structural and physical properties of pharmaceutical compounds. The structural and physicochemical properties of compounds can be calculated by molecular fingerprints or molecular descriptors, which have been used for drug development and toxicity prediction [10]. Moreover, various QSAR models for predicting hepatotoxicity have been reported, most of which use machine learning methods, but their prediction performances are still unsatisfactory [11]. Ekins et al. adopted Bayesian classifier with 295 compounds as training set and 237 compounds as test set, and obtained an accuracy of 57%–59% on the training set and an accuracy of 60% on the test set [12]. Liew et al. proposed an ensemble classifier based on support vector machine (SVM) and k-nearest neighbor (kNN), which achieved an overall accuracy of 63.8% with five-fold cross-validation on 1087 compounds, and an accuracy of 75.0% on an additional verification dataset of 120 compounds [6].

This work implemented an improved strategy to develop a QSAR model for predicting DILI in humans, with Food and Drug Administration (FDA)-approved drug labeling data [13]. The FDA-approved drug labeling is the authoritative document which comprehensively summarizes drug safety information from clinical trials, post-marketing surveillance, and literature publications. The set of drugs was recommended as the standard list for developing DILI predictive models [14]. Then, 12 types of molecular fingerprints and 7 molecular descriptors were used. Moreover, five machine learning methods were adopted to predict the hepatotoxicity of compounds. Finally, an ensemble system was built combining various molecular fingerprints, molecular descriptor subsets, and various models generated by machine learning methods. Our model is primarily used for filtering out compounds with potential hepatotoxic risks in the early stages of drug development before the clinical phase.

## 2. Results

In this study, 12 molecular fingerprints and 9 machine learning methods were used to predict drug-induced liver injury, generating 108 base classifiers, which were evaluated by 5-fold cross-validation [15]. From the 450 compounds of this work, 50 compounds were randomly extracted from the data set as an independent test data set, and other compounds as a training data set. The cross-validation process was then repeated 5 times, where each of the 5 subsamples was used exactly once as the training data. In addition, the whole process was repeated 1000 times in order to reduce the randomness of predictions and accurately evaluate the performance of the model. 

### 2.1. Parameter Selection for the Proposed Method

First, good classifiers with certain descriptors should be obtained by performing the 108 base classifiers on the whole training dataset. The aim is to select top classifiers, which are then used to build our proposed method. The results are shown in Table 1. It can be seen that 9 classifiers and 12 molecular fingerprints together produce 108 accuracies. From the Table 1, a set of top 5 classifiers were obtained for each of the 12 molecular fingerprints. Then, for each base classifier, the number of obtained top 5 classifiers with different fingerprint descriptor were counted. The number for XGBT is 10, 11 for CatBT, 10 for RF, 9 for GDBT, 9 for LGBT, 6 for ExtraTrees, 2 for AdaBT, 2 for LR, and 0 for SVM. Therefore, top 5 base classifiers are XGBT, CatBT, RF, GDBT, and LGBT, which are then used to build ensemble vote classifier. More details of performance information for 108 basic classifiers can be referred to supplementary 2.

After the top 5 classifiers were selected, the average accuracy of the five basic classifiers were then calculated for each fingerprint. The details of the selected top 5 classifiers can be referred to supplementary 3. Table 2 lists the average accuracy for each fingerprint, whose detailed information can be seen in supplementary 4.

**Figure 2:**
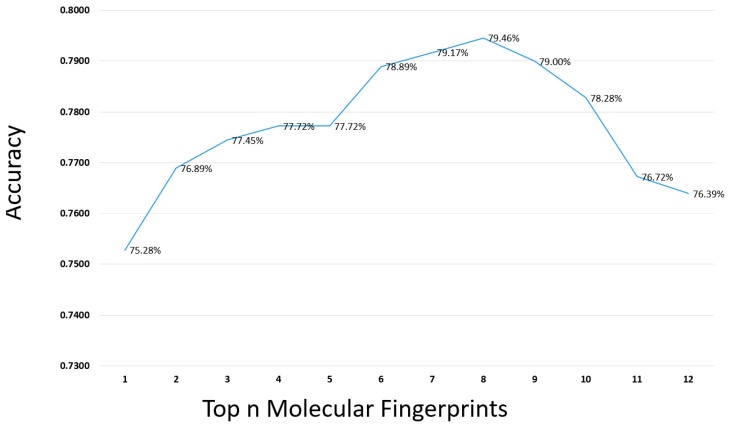
The selection of top *n* molecular fingerprints from the 12 molecular fingerprints by top 5 classifier.

The 12 molecular fingerprints were sorted in terms of the average accuracy of top 5 classifiers in Table 2. First, the accuracy of the top 5 classifiers with the top 1 molecular fingerprints feature (ExtendedFP) was selected. Then, another molecular fingerprint feature from top to low in Table 2 was added each time, and then prediction results were achieved for the combined fingerprints. The process was ran20 times and the average performance was obtained, as shown in Figure 2. From the Figure 2, we can see that when the number of molecular fingerprints increased to eight, the maximum accuracy was obtained, indicating that these eight molecular fingerprints are very important for the prediction of drug-induced liver injury. Therefore, the first eight molecular fingerprints were considered for the next step. The detailed information of finding the top fingerprints can be seen in Supplementary 5.

**Figure 3:**
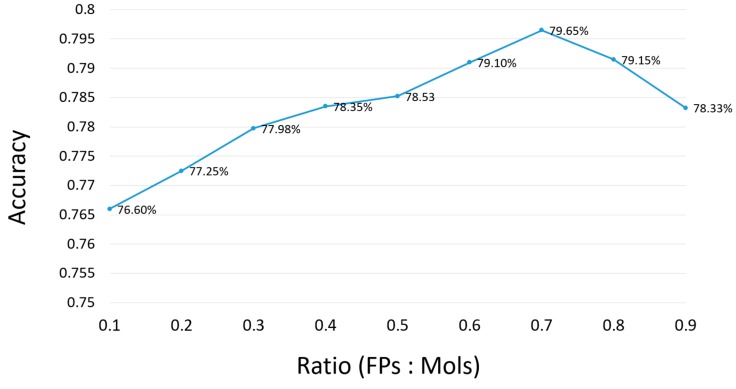
Select best weight ratio of fingerprints to molecular descriptors. Abbreviations: FPs, fingerprints; Mols, molecular descriptors.

In order to improve the accuracy of the model, seven key physicochemical properties were used, which were widely adopted in chemical toxicity prediction [6,11,12]. Then the weight of the molecular descriptors and fingerprints was further investigated. Figure 3 illustrates the accuracy of model in terms of the threshold of the weight. From Figure 3, it can be seen that the best weight is 7:3 to tradeoff molecular fingerprints and molecular descriptors, The detailed information can be seen in Supplementary 6.

### 2.2. Performance of the Proposed Method

To integrate the advantages of various algorithms and fingerprints, several combination models were built based on 108 base classifiers. First, 108 classifiers were sorted by accuracy. Then the top *n* base classifiers with the highest accuracies were selected, whose average prediction probability was used to re-predict liver toxicity. In this process, an optimal integration model of five base classifiers was obtained. As expected, the ensemble model obtained higher accuracy than any base classifier. In addition, almost the ensemble model performed better than single base classifiers in both sensitive and specific. Due to the combination of diversity and independence of different models, the ensemble model achieved better prediction performance. The best ensemble model consists of five base classifiers: GDBT, XGBT, RF, LGBT, and CatBT, which results by 5-fold cross-validation achieved an accuracy of 77.25%, an SE of 64.38%, a SP of 85.83%, an AUC of 75.10%. The experimental results showed that the ensemble method can improve the performance of hepatotoxicity prediction.

Experimental can objectively reflect the ability of the model to predict hepatotoxicity of compound. On the independent test set the model achieved an accuracy of 81.67%, an SE of 64.55%, an SP of 96.15%, an AUC of 80.35%, this result showed that our integrated model can effectively and stably predict the liver damage of drugs. Table 3 lists performance comparison of 9 algorithms with 12 molecular fingerprints on test set, and the detailed information can be seen in Supplementary 7.

## 3. Discussion

### 3.1. Comparison with Previous Methods on Different Datasets

Many methods have been developed for predicting drug-induced liver damage [9,12,16]. Table 2 lists the performance comparison of several methods on different datasets. From Table 4, although the choices of data sets, data preprocessing and feature selection are different, the higher accuracy of our model indicated that our model is more advantageous for drug-induced liver injury than other models. Moreover, our model obtained a relatively high SP. Specificity reflects the correct identification of drugs without drug-induced liver damage, which is an important indicator for evaluating drug-induced liver injury classifiers. 

### 3.2. Comparison with Previous Models on the Same Dataset

To make fair comparison with other methods, experiments of our method were implemented on the same dataset of literature [14], which used a dataset [13] of 451 compounds, containing 183 most-DILI drugs and 268 no-DILI drugs. Our method used the same dataset [13], where the difference is in that one most-DILI drug was ignored because in the DILIrank dataset, the most-DILI drug does not have PubChem_CID. Therefore, our method used the dataset of 450 compounds, containing 182 most-DILI drugs and 268 no-DILI drugs.

In literature [14], authors adopted PaDEL-Descriptor software to obtain molecular descriptors. and then a pattern recognition algorithm DF (http://www.fda.gov/ScienceResearch/BioinformaticsTools/DecisionForest/default.htm) to build a DILI risk prediction model. Moreover, authors performed 5-fold cross-validations to estimate the model on the dataset, by running the model 1000 iterations. Finally, the model achieved average prediction accuracy, sensitivity, specificity and Matthews correlation coefficient (MCC) of 72.9%, 62.8%, 79.8%, and 0.432, respectively. Our method was implemented on the same conditions of literature [14] and yielded average prediction accuracy, sensitivity, specificity and Matthews correlation coefficient (MCC) of 76.9%, 62.2%, 87.0%, and 0.514, respectively. The prediction comparison is listed in Table 5. From Table 5, we can get that our method performs better than Decision Forest [14] in accuracy, specificity and Matthews correlation coefficient (MCC).

### 3.3. Molecular Descriptors and Fingerprints related to Hepatotoxicity 

Molecular fingerprints are important features for drug-induced liver injury, which were calculated using PaDEL-Descriptor software for compounds. PaDEL-Descriptor software can create a total of 12 molecular fingerprints, which outputs different fingerprint values for different compounds. The data provided by the US Food and Drug Administration (FDA) was used to determine which molecular fingerprints are more relevant for drug-induced liver injury. We calculate the exact value of a single fingerprint and can determine this value for the field of drug-induced liver injury. Correlation size, we can get the correlation between 12 molecular fingerprints and DILI from Table 2. We can see that the fingerprint with the largest correlation for DILI is ExtendedFP, followed by KRFP, and the least relevant is nAP2DFP. The information is important to drug developers.

From previous literature, it can be found that molecular descriptors are related to toxicity, which have also been used for the prediction of drug-induced liver injury. The optimal weight of fingerprints and molecular descriptors is 0.7, which indicates that fingerprints are more advantageous for predicting drug-induced liver damage. Compared with the molecular fingerprint, the molecular descriptor has a small contribution to the whole model, and the weight is only 0.3. 

### 3.4. Applicability Domain of Model

The similarity measurement of our ensemble model is based on the transformation of chemical information, represented by molecular symbols of compound, into useful mathematical numbers. Description file of compound involving two-dimensional chemical structure was extracted from the PubChem database by CID number. Then the structure information of the compound was encoded by molecular descriptor [17]. The main difference between this and other methods is in that the mathematical parameters can be used to characterize the molecular descriptors, and to calculate the correlation between the descriptor values and biological activity [18]. Therefore, our model is suitable for early drug design, particularly for screening and predicting compounds of drug-induced liver injury.

## 4. Materials and Methods

### 4.1. Data Preparation

To develop reliable models for predicting human DILI risk, a set of 450 drugs was used which was extracted from the DILIrank dataset [13], which containing 192 most-DILI and 312 no-DILI risk drugs. In order to obtain better prediction, structure description file (SDF) was used, which was obtained using PubChem CID number provided by DILIrank, for building our proposed model. The two-dimensional (2D) chemical structure description file of the 450 drugs were generated from https://www.ncbi.nlm.nih.gov/pccompound through the PubChem CID number. Finally, 450 drugs were obtained containing 182 positive samples and 268 negative samples with SDF files. Details of the dataset are provided in Supplementary 1. We randomly divided 450 samples into nine equal parts, eight of which were training sets and the rest one was an independent test set.

### 4.2. Calculation of Molecular Fingerprints

Twelve types of molecular fingerprints were used to indicate the chemical structural characteristics of compounds. Table 6 summarizes the details of these molecular fingerprints. Molecular descriptors are quantitative representations of structural and physicochemical features of molecules. Herein, 7 key physicochemical properties were adopted, including Ghose–Crippen log K_ow_ (AlogP), molecular weight (MW), the number of aromatic rings (nAR), the number of hydrogen bond acceptors (nHBA), the number of hydrogen bond donors (nHBD), the number of rotatable bonds (nRTB), and the number of rings (nR), which were widely adopted in chemical toxicity prediction [19,20,21]. These properties formed as a set of molecular descriptors and were used as a part of the weight for model building. All molecular fingerprints were calculated by PaDEL-Descriptor software (version 2.21) using the SDF files of all compounds [22].

### 4.3. Feature Selection

Features selection is an important step for the construction of model. In order to improve the prediction accuracy of the model, some unimportant features were usually removed. In this study, the accuracies of 12 molecular fingerprints through 9 basic classifiers were investigated, which were sorted and those unimportant molecular fingerprints were removed. As a result, 8 molecular fingerprints were obtained.

### 4.4. Model Building

#### 4.4.1. Base Classifiers

The logistic regression (LR), support vector machine (SVM), random forest (RF), gradient boosting (GDBT), Adaboost (AdaBT), Xgboost (XGBT), ExtraTrees, Lightgbm (LGBT), and Catboost (CatBT) were adopted as base classifiers for our ensemble system. LR fits the data into a logit function, whose purpose of performing logistic regression is to minimize the error between the tag value of training data and the predicted value. SVM [23] maps the features of the input data to higher dimensional spaces through several kernel functions to separate positive and negative instances. In this study, a radial basis kernel function was used to construct the SVM model. GDBT [24] makes use of decision trees as the base classifiers, which can apply steepest descent to minimize the loss function on the training data. Adaboost is to train different classifiers (weak classifiers) for the same training set, and then combine these weak classifiers to form a stronger final classifier (strong classifier) [25]. During training, each weak classifier is trained in turn and their weight values are obtained, which are constructed according to their accuracy, i.e., the weak classifier with higher accuracy will be assigned greater weight. Xgboost uses clever penalization of the individual trees, and the trees are consequently allowed to have varying number of terminal nodes [26,27]. RF [28] is an ensemble learning method that operates through constructing a multitude of decision trees at training time and outputting the class, which is the mode of the classes or mean prediction of the individual trees. ExtraTrees [29] is essentially consists of randomizing strongly both attribute and cut-point choice while splitting a tree node. Lightgbm [30] is mainly based on a single machine to use as much data as possible without sacrificing speed, based on Gradient-based One-Side Sampling (GOSS) and Exclusive Feature Bundling (EFB). CatBoost (categorical boosting) [31] is a library of gradient lifting algorithms that first randomly sorts all samples and then takes a value for each of the categorical features, and the feature of each sample is converted to a numeric type. 

#### 4.4.2. Ensemble Model

Ensemble vote classifier [32,33] combined similar or conceptually different machine learning classifiers and tried to obtain better predictive performance than individual classifier alone [34,35,36], via majority or plurality voting. In our study, soft voting was implemented, which predicted test instances by averaging the class-probabilities of different classifiers. In particular, ensemble learning methods tended to produce better results because of the significant differences between different classifier models, which have been widely used in many fields, including toxicity prediction [6].

**Figure 4:**
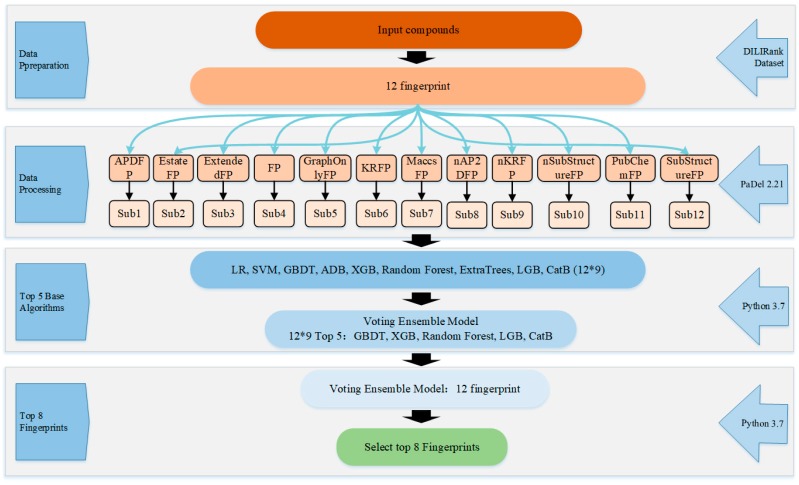
Flowchart of the ensemble classifier system with top 5 classifiers and top 8 fingerprint filters.

To build the ensemble system, first, dataset with instances encoded by each fingerprint was input into each base type of classifiers. Therefore, 108 classifiers were built with 12 molecular fingerprints based on 9 machine learning algorithms. To vote among the nine base classifiers, the top 5 base classifiers with better prediction performance were obtained, similarly, the top 8 of 12 fingerprints were obtained. Figure 4 shows the flowchart of the ensemble classifier system.

**Figure 5:**
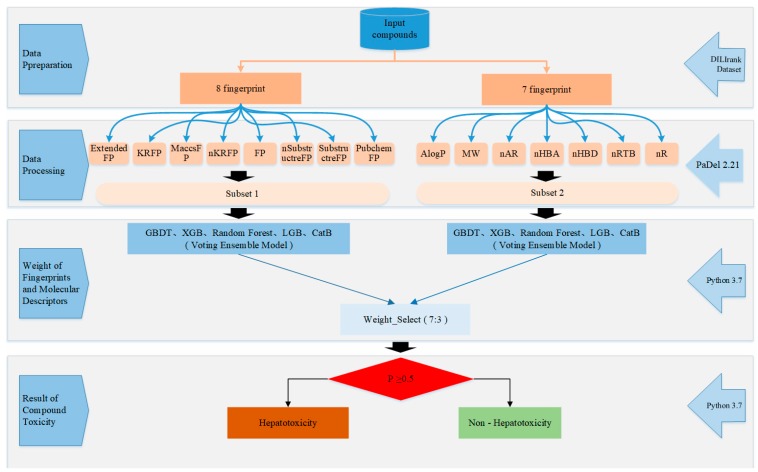
Flowchart of the ensemble model.

Figure 5 shows the flowchart of ensemble model. First, the top 8 molecular fingerprints were used as a subset, and 7 molecular descriptors as the second subset, using an ensemble vote classifier method to calculate the weight ratio of molecular fingerprints and molecular descriptors. The default threshold for our model was set to 0.5, which means that compounds with a hepatotoxic probability greater than 0.5 will be classified as hepatotoxic, otherwise, nonhepatotoxicants.

### 4.5. Performance Evaluation

For the purpose of making our model more reliable, randomized testing was used, which ensures the robustness of the predictive model [17,37]. Because the distribution of our data is random, the matrix of original independent variable was generated randomly. The prediction model was running several times and average performance was obtained, which is to ensure that the model involving randomly generated independent variable matrix is reliable.

Four indicators were used to assess the predictive performance of model [38]: accuracy (ACC), the overall prediction accuracy of hepatotoxicants and nonhepatotoxicants; sensitivity (SE), the prediction accuracy for hepatotoxicants; specificity (SP), the prediction accuracy for nonhepatotoxicants; the area under the receiver-operating characteristic curve (AUC). These indicators were calculated as follows:

(1) $$\mathrm{ACC}=\frac{\mathrm{TP}+\mathrm{TN}}{\mathrm{TP}+\mathrm{TN}+\mathrm{FN}+\mathrm{FP}}\times 100\%$$

(2) $$\mathrm{SE}=\frac{\mathrm{TP}}{\mathrm{TP}+\mathrm{FN}}\times 100\%$$

(3) $$\mathrm{SP}=\frac{\mathrm{TN}}{\mathrm{TN}+\mathrm{FP}}\times 100\%$$

where true positive (TP) is the number of the hepatotoxicants that are correctly predicted, true negatives (TN) is the number of the nonhepatotoxicants that are correctly predicted, false positive (FP) is the number of the nonhepatotoxicants that are wrongly predicted as hepatotoxicants, and false negative (FN) is the number of the hepatotoxicants that are wrongly predicted as nonhepatotoxicants.

The AUC (area under curve) was calculated for the prediction ability of the model. ROC (receiver operating characteristic curve) graphs are two-dimensional graphs that plotted curves of TP rate with respect of FP rate. ROC graph depicts relative tradeoffs between benefits (true positives) and costs (false positives) [39]. The value of AUC is between 0.1 and 1, which can be used to visually evaluate the quality of the classifier, i.e., the larger value of AUC illustrates that the model is better. 

## 5. Conclusion

This paper adopted nine machine learning classifiers and 12 molecular fingerprints to predict compounds, selected five top-level base classifiers to build an ensemble model, and finally got eight molecular fingerprints with relatively large correlations with DILI. Our integrated model is superior to a single classifier model. In addition, we also found that molecular descriptors related to drug-induced liver injury from the literature can improve the performance of the model, and get the best weight of molecular descriptors and fingerprints for DILI. The 5-fold cross-validation accuracy of the model is 77.25%, 64.38%, for SE, 85.83% for SP, and 75.10% for AUC. Our model also achieved good results on independent test sets with an accuracy of 81.67%, SE of 64.55%, SP of 96.15%, and AUC of 80.35%. Experimental results indicated that our ensemble model performs good in predicting drug-induced liver injury, and outperforms other previous methods.

## Figures and Tables

**Table 1:** Performance Comparison of Base Classifiers on the Whole Training Dataset.

No	Descriptor	Base Classifier								
		LR	SVM	GDBT	AdaBT	XGBT	RF	ExtraTrees	LGBT	CatBT
1	AP2DFP	**0.7222 ^#^**	0.6978	**0.7322**	**0.7233**	**0.7222**	0.7067	0.6944	0.6911	**0.7278**
2	Estate FP	0.7078	0.7044	**0.7278**	0.6811	**0.7322**	**0.7433**	0.7211	**0.7233**	**0.7289**
3	ExtendedFP	**0.7789**	0.7322	0.7511	0.7355	**0.7556**	**0.7778**	0.7333	**0.7689**	**0.7933**
4	FP	0.7133	0.6878	**0.7500**	0.7111	**0.7478**	**0.7444**	0.7056	**0.7422**	**0.7811**
5	GraphOnlyFP	0.7067	0.6689	**0.7267**	0.7056	**0.7211**	**0.7345**	0.7089	**0.7178**	**0.7151**
6	KRFP	0.7500	0.7344	0.7522	0.7211	**0.7578**	**0.7811**	**0.7622**	**0.7611**	**0.7789**
7	MaccsFP	0.7300	0.7045	0.7389	0.7256	**0.7578**	**0.7722**	**0.7456**	**0.7589**	**0.7722**
8	nAP2DFP	0.6933	0.6822	**0.7144**	0.6889	**0.7078**	**0.7044**	**0.7055**	**0.7033**	0.7033
9	nKRFP	0.7522	0.7056	**0.7589**	0.7356	0.7544	**0.7733**	**0.7567**	**0.7545**	**0.7578**
10	nSubstructreFP	0.7111	0.7033	**0.7644**	0.7111	**0.7378**	**0.77**	**0.7355**	0.7289	**0.7633**
11	PubchemFP	0.7278	0.6956	**0.7522**	0.7100	**0.7389**	**0.75**	0.7167	**0.7322**	**0.7589**
12	SubstructreFP	0.7300	0.7267	**0.7500**	**0.7378**	0.7244	**0.7911**	**0.7700**	0.7189	**0.7622**
	Number (Top 5)	**2**	**0**	**9**	**2**	**10**	**11**	**6**	**9**	**11**

**^#^** The bolt numbers in each row denote the top 5 classifiers with the specific fingerprint descriptor.

**Table 2:** Sorted Average Accuracies of the Top 5 Classifiers with Respect of Different Fingerprints.

NO	Fingerprint	Average Accuracy
**3**	ExtendedFP	0.7693
**6**	KRFP	0.7662
**7**	MaccsFP	0.7600
**9**	nKRFP	0.7598
**4**	FP	0.7531
**10**	nSubstructreFP	0.7529
**12**	SubstructreFP	0.7493
**11**	PubchemFP	0.7464
**2**	EStateFP	0.7311
**5**	GraphOnlyFP	0.7230
**1**	AP2DFP	0.7160
**8**	nAP2DFP	0.7067

**Table 3:** Performance Comparison of Base Classifiers on the Test Dataset.

Algorithms/Fingerprints	LR	SVC	GBDT	ADB	XGB	Random Forest	Extra Trees	LGB	CatB
AP2DFP	0.7000	0.6600	0.6200	0.5800	0.6800	0.6640	0.6480	0.6800	0.6360
Estate FP	0.6600	0.6800	0.7000	0.6800	0.7000	0.6880	0.7200	0.7000	0.7160
ExtendedFP	0.7800	0.7400	0.7000	0.7600	0.7400	0.7480	0.7360	0.6600	0.7800
FP	0.6600	0.7000	0.7440	0.6600	0.7000	0.7240	0.6760	0.7400	0.7320
GraphOnlyFP	0.6000	0.6000	0.6720	0.6400	0.7200	0.6840	0.6720	0.7200	0.6960
KRFP	0.7200	0.6400	0.7240	0.6600	0.7000	0.7840	0.7600	0.7400	0.7520
MaccsFP	0.7600	0.7200	0.7200	0.7000	0.7200	0.7520	0.7040	0.7200	0.7360
nAP2DFP	0.6600	0.6200	0.6360	0.6000	0.6600	0.7040	0.6600	0.6400	0.6880
nKRFP	0.6600	0.6200	0.7160	0.7400	0.7200	0.7520	0.7480	0.7000	0.7320
nSubstructreFP	0.7200	0.6400	0.6400	0.5400	0.6400	0.5920	0.6280	0.6200	0.6200
PubchemFP	0.7200	0.7600	0.7040	0.6200	0.7400	0.7760	0.7360	0.6600	0.7480
SubstructreFP	0.7400	0.7600	0.7200	0.7200	0.7000	0.7360	0.7280	0.6800	0.7440

**Table 4:** Performance Comparison of Several Hepatotoxicity Prediction Models.

Model Name	No. of Compounds	Test Method	Q (%)	SE (%)	SP (%)	AUC(%)
Bayesian [12]	295	10-fold CV×100	58.5	52.8	65.5	62.0
Decision Forest [9]	197	10-fold CV×2000	69.7	57.8	77.9	–
Naive Bayesian [16]	420	Test set	72.6	72.5	72.7	–
Our Method	450	5-fold CV×1000	77.25	64.38	85.83	75.10
		Test set	81.67	64.55	96.15	80.35

Abbreviations: Q: accuracy; SE: sensitivity; SP: specificity; AUC: area under the curve.

**Table 5:** Performance Comparison of Previous Models.

Model Name	No. of Compounds	Test Method	Q (%)	SE (%)	SP (%)	AUC (%)	MCC (%)
Decision Forest [14]	451	5-fold CV	72.9	62.8	79.8	–	51.4
Our Method	450	5-fold CV	76.9	62.2	87.0	74.6	43.2

Abbreviations: Q, accuracy; SE, sensitivity; SP, specificity; AUC, area under the curve; MCC, Matthews correlation coefficient.

**Table 6:** Summary of the 12 Types of Molecular Fingerprints.

Fingerprint Type	Abbreviation	Pattern Type	Size (bits)
CDK	FP	Hash fingerprints	1024
CDK Extended	ExtendedFP	Hash fingerprints	1024
CDK GraphOnly	GraphOnlyFP	Hash fingerprints	1024
Estate	EstateFP	Structural features	79
MACCS	MaccsFP	Structural features	166
Pubchem	PubchemFP	Structural features	881
Substructure	Substructure	Structural features	307
Substructure Count	nSubstructure	Structural features count	307
Klekota-Roth	KRFP	Structural features	4860
Klekota-Roth Count	nKRFP	Structural features count	4860
2D Atom Pairs	AP2D	Structural features	780
2D Atom Pairs Count	nAP2DC	Structural features count	780

## Supplementary Materials

Supplementary materials can be found at https://www.mdpi.com/1422-0067/20/17/4106/s1.
