# Supplementary material for: In Silico Prediction of Drug-Induced Liver Injury Based on Ensemble Classifier Method
Source: Int J Mol Sci. 2019 Aug 22;20(17):4106. doi: 10.3390/ijms20174106 (PMC6747689; doi:10.3390/ijms20174106)
Supplement: Supplementary file 1 [file ijms-20-04106-s001.zip › Supplementary caption.docx]

**Supplementary 1:** The detailed SDF files for the used dataset. The files were generated from https://www.ncbi.nlm.nih.gov/pccompound through the PubChem CID number.

**Supplementary 2:** The more details of performance information for 108 base classifiers. The 108 classifiers were built with 12 molecular fingerprints based on 9 machine learning algorithms.

**Supplementary 3:** The numbers of obtained top 5 classifiers. The top 5 classifiers were selected for each fingerprint descriptor and the numbers of obtained top 5 classifiers for each base classifier were counted, then the best base classifiers were selected.

**Supplementary 4:** The sheet 1 shows average accuracy for each fingerprint over the top 5 base classifiers; the sheet 2 shows the rank of fingerprints with respect of average accuracy of top 5 classifiers.

**Supplementary 5:** Performance comparison of the top *n* molecular fingerprints for running 20 times. First, the accuracy of the top 5 classifiers with the top 1 molecular fingerprints feature (ExtendedFP) was selected. Then, another fingerprint feature from top to low in Table 3 was added each time, and then prediction results were achieved for the combined fingerprints. The process was ran20 times and the average perfomance was obtained.

**Supplementary 6:** The weights of molecular fingerprints and molecular descriptors for running 10 times. The average performance can be further obtained.

**Supplementary 7:** The sheet 1 shows the performance of 9 classifiers with 12 molecular fingerprints for running five times on the test set; the sheet 2 shows the average accuracy for the 12 fingerprints on the test set.
